# Horizon scanning: what next for bovine TB control in England?

**DOI:** 10.1186/s13620-023-00242-z

**Published:** 2023-07-31

**Authors:** James McCormack

**Affiliations:** Animal and Plant Health Agency, London, UK

**Keywords:** Bovine tuberculosis, Horizon scanning, Knowledge based trading, Whole genome sequencing, DIVA test, Cattle vaccination, Badger culling

## Abstract

England is currently in year nine of its 25-year strategy to achieve TB freedom. This talk will speculate on what new tools and approaches could be introduced in the future to help us achieve our goal.

Using Defra’s response to the independent review of its TB programme as a starting point and building on the plenary talk by the UK CVO, I will look at some opportunities that could arise under the different aspects of the response.

Firstly, how best to help farmers reduce their herd TB risk through better informed purchasing decisions will be considered, including looking at the recent publication of the health ratings for every cattle herd in England.

Cattle vaccination, and its associated DIVA test could be the biggest change in Tb control in England in many years while the related development of a molecularly defined tuberculin which could become the default testing reagent.

Advances in whole genome sequencing will allow us to sequence the genome of *M.bovis* isolated from most infected herds in England and these data could unlock a variety of opportunities from tracing the spread of infection to ground-truthing the efficacy of testing and epidemiological assessment of breakdowns.

Finally, the move to vaccination as the primary way of controlling TB in badgers with culling used very sparingly will be considered using a case study of how a targeted badger cull successfully removed infection from an area in Cumbria and enabled the switch to vaccination.

## Introduction

**Fig. 1:**
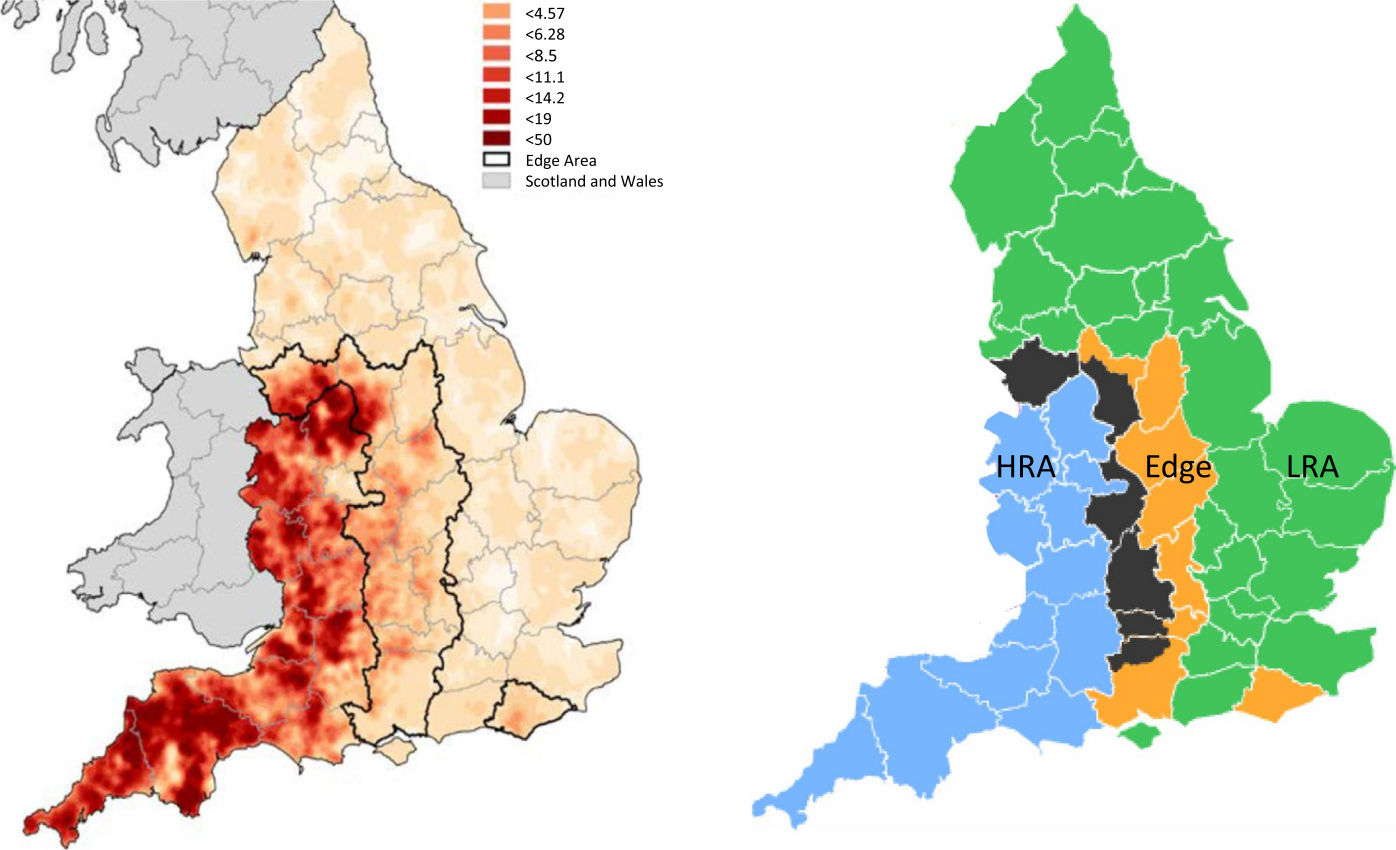
Herd incidence in England 2020 (left) the division of England into High-Risk Area (HRA) (blue), Low-Risk Area (LRA) (green) and Edge Area. The Edge Area is further subdivided into 6 monthly testing area (black) and annual testing area (orange)

Defra is the government department responsible for bovine TB control in England. In 2014 it published [1] its 25-year strategy with a goal of achieving officially TB free status for England by 2038. As part of that strategy England was subdivided into three zones based on herd incidence (Fig. 1); the High-Risk Area (HRA) the Low-Risk Area (LRA) and the Edge area (which was later subdivided).

Since 2014 there has been a steady increase in control measures in cattle with increases in the frequency of surveillance testing in cattle, the introduction of pre-movement testing and increased use of interferon-gamma testing. Badger culling was allowed under licence in 2013 and expanded rapidly from 2016 to the present.

**Fig. 2:**
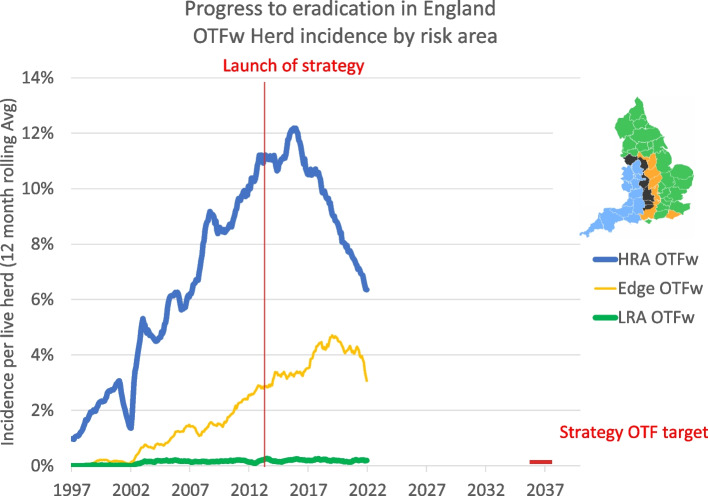
OTFw incidence in the three TB Areas of England. The OTF target is a horizontal red bar. The launch of the strategy is indicated by a vertical red line

So what progress has been made since 2013? Figure 2 shows Officially TB Free withdrawn (OTFw) herd incidence by risk area [2]. OTFw herds are herds where at least one animal tests positive and has visible lesions and/or *M.bovis* isolated post-mortem. Incidence needs to be at 0.1–0.2% by 2038 in order for England to be eligible for OTF status. In the HRA we have seen 20 years of increase in incidence up to 2017 but a decline over the last five years. The Edge area has seen a steady increase until the last year or so. LRA incidence is low and stable and is close to OTF status. So we have a long way to go but we are moving slowly in the right direction.

Defra’s TB strategy was independently reviewed [3] in November 2018 by Prof. Charles Godfray and colleagues and the government responded [4] in March 2020 laying out the five outputs of the response. The key goal remains declaring England officially free of TB by 2038 and work will focus on the following five areas.

- Acceleration of work to develop a Cattle vaccine and DIVA test
- Changing the strategy for preventing the spread of TB from wildlife
- Improving diagnostics surveillance and epidemiology
- Incentivising industry behaviours
- Creating a partnership with government industry and other stakeholders

## Outline

Today I want to look ahead at what is on the horizon in terms of innovation. Bearing in mind the need to balance cutting edge technology with practical tools that can be deployed at scale, cost effectively to improve the overall efficiency of the TB control system.

**Fig. 3:**
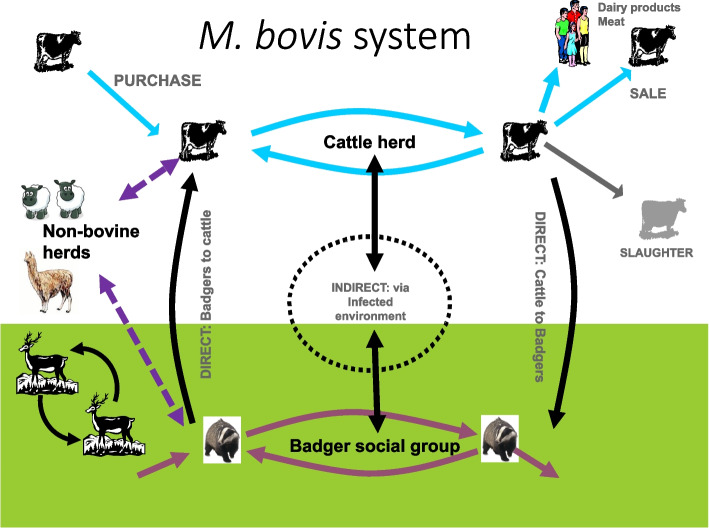
Depiction of the routes *M.bovis* spreads between cattle and badgers and other species

**Fig. 4:**
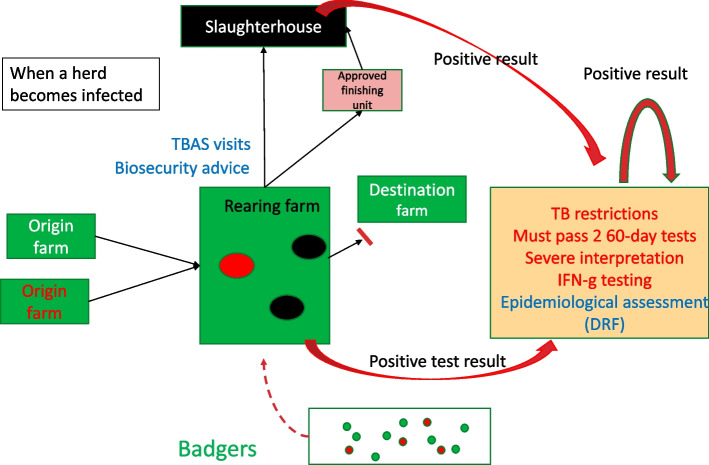
The effect of TB controls on infected farms. Summarising the testing regime when infection is detected (orange box) and the prohibition on onward movements (red bar)

This brings us to the *M.bovis* system where we consider how *M.bovis* circulates within and between badgers and cattle (Fig. 3). Each of the pathways on this system is a possible route to reduce through new or stronger control measures. However, we normally don’t deal with the bacteria directly but rather with how it affects cattle and farmers so we also need to consider the farming and TB disease control system (Fig. 4). Different farm types are impacted by TB control measures to very different extents depending on their trading patterns.

Cattle controls are comprised of both compulsory measures and advice. When TB is detected the herd is placed under movement restrictions and usually all animals must pass two short interval skins tests at severe interpretation and the herd will often undergo interferon gamma testing. Herds may also undergo in-depth epidemiological assessment to try and ascertain the most likely route of transmission. In many parts of England badgers are a reservoir of disease and the two approaches taken to control this risk in England are badger culling and badger vaccination.

So against this background of the disease and its controls and bearing in mind the government response to the Godfray review. I want to focus on different Innovations that could improve the TB control system.

- Knowledge based trading
- Whole genome sequencing
- Cattle Vaccine + DIVA test
- Molecularly defined tuberculin
- Targeted badger culling

This is a speculative discussion about only some of the potential approaches we could take. This is not an announcement of agreed policy and indeed I have more questions than answers.

### Knowledge based trading

Purchasing cattle is a significant route of introducing infection into a herd [5–7]. Therefore, one goal of our strategy is to reduce this aspect of disease spread by providing farmers with more information so that they can understand and incorporate knowledge of disease risk into their purchasing decisions. This is termed Knowledge Based Trading (KBT). Herd history is a significant risk factor, for example in the HRA, 57% of the breakdowns in 2020 came from the 27% of herds who had TB in the last 3 years. This can be analysed further and time since last breakdowns is quite a good predictor of future risk. This has been converted into a “health score” and updated on the publicly available ibtb website www.ibtb.co.uk which allows farmers to look at every herd in England and see how long since their last TB breakdowns and how long that breakdown lasted.

We would like to go further to incorporate more KBT information in the Livestock Transformation Programme. This would include an online portal to provide information about the health status of cattle when they are traded or otherwise moved from herd to herd. The current phase is developing a portal to communicate information on TB and Bovine Viral Diarrhoea both at the level of the individual animals and also of the herds they have been in during their life. This would provide herd owners with key information to enable them to understand and manage the bovine TB and BVD disease risks posed by the cattle they may wish to move into their herd.

### Questions remaining on KBT

- Can we develop the IT system with a sufficiently high level of reliability and accuracy?
- Is simply providing this information sufficient?
- What effect on trading patterns could this approach have?
- What impact would this have on the epidemic?
- What impact will this have on the industry where many farmers need to buy and/or sell regularly?
- What further incentives on cattle movements could be introduced?

### Whole genome sequencing

I now want to talk about Whole Genome Sequencing (WGS). To recap, WGS is a molecular method that characterizes the complete DNA sequence of an organism’s genome. In recent years it has become faster and cost-efficient using multiplexing approaches. APHA currently sequences all *M.bovis* isolates in GB and over 15,000 M*.bovis* isolates have been sequenced so far.

Analysis of these 15,000 samples has shown the High-level Population Structure of *M.bovis* in GB with 30 distinct lineages (clades) identified. The majority of these lineages are endemic to defined geographic locations of England or Wales.

The end–to-end process from sequencing to defining clade is now accredited to ISO 17025 standard and the old approach of genotyping using spoligotyping and VNTR has been discontinued. Currently we are working on phase 2 which will provide validated phylogenetic analysis combined with GIS data to deliver a functional web-based GIS system available for use by all user groups.

In this conference we have heard several talks looking at how WGS can be used to estimate the relative transmission rates between and within cattle and wildlife reservoir species, but I would like to look at an example of how we are using it to understand individual cattle breakdowns. Exploiting the fact that WGS allows much greater discrimination than genotyping**.**

Recently there was a case of an animal which tested positive on a farm near Luton in the Low-risk area. The breakdown farm had a history of purchasing from the high-risk area whereas the animal had been on two other farms, both with clear TB testing histories. The initial conclusion without WGS was that the infection on the Luton farm came from another bought-in animal from the HRA. However, WGS allowed us to look at the closest genetically related isolates to that in the infected animal. This showed that all the related isolates came from a geographical cluster of breakdowns near the second farm the infected animal had been on. Therefore, this strongly suggests that the second farm is the most likely source of the infection despite the animal’s clear test on that farm and the farm’s lack of a TB history.

### WGS opportunities and challenges

#### Opportunities

- Identifying Local origin vs imported cases.
- Developing cluster-based approaches depending on whether a cluster of infection is due to local spread or multiple imported cases.
- Identifying missed tests such as the second farm in the example above—such false negatives give insights into test sensitivity, disease transmission and possibly problematic testers.
- Linking cattle movements with WGS could more clearly show farmers where their breakdown came from which will help Knowledge Based Trading.
- Improve epidemiological assessments and could act as a check on initial source attribution

### Challenges

- How many breakdowns will be simple stories that can be easily explained?
- Timing gap- the first isolate sequenced may not be the source and it can take time to get enough samples to have a clear picture.
- Some herds have multiple independent infections although usually only 1 sample is cultured and sequenced.

### Cattle vaccine, diva test and molecularly defined tuberculin

Our candidate vaccine is CattleBCG. BCG produces a spectrum of protection whereby some cattle are fully protected, some partially protected, and some are not protected at all. Estimates of efficacy vary from ~ 50% in a recent meta-analysis [8], although a large New Zealand field trial [9] suggested an overall protective efficacy of 85%.

However, BCG cross-reacts with the official tuberculin skin test generating up to 80% false positives and largely for this reason it is not currently an authorised veterinary medicine. To overcome this APHA have developed a candidate skin test, DST-F, to detect infected animals among vaccinated animals (DIVA test). This consists of a fusion protein containing three antigens from *M.bovis* that BCG vaccinated animals do not react to.

Developing a deployable cattle TB vaccine means securing UK marketing authorisations from the UK’s Veterinary Medicines Directorate (VMD) for both CattleBCG and DST-F. The VMD has granted APHA permission to conduct field trials to generate data for UK marketing authorisations. The World Organisation for Animal Health (WOAH/OMSA) envisages that vaccination could be applied in combination with DIVA tests once these have been fully validated. Therefore, to deploy CattleBCG without trade implications we will also need WOAH validation of DST-F.

Currently field trials are underway in order to apply for marketing authorisation of the vaccine and DIVA test. Phase 1 started in June 2021 and is assessing specificity and safety of DST-F in the Low-Risk Area. Phase 2, which is dependent on the outcomes of Phase 1, will look at the safety of CattleBCG and specificity of DST-F. There are other dependencies for a successful licensing including recruiting a Contract Manufacturing Organisation to produce DST-F to Good Manufacturing Practice, and on a research project looking at the duration of immunity of CattleBCG.

If Cattle BCG does receive marketing authorisation the next issue will be to consider how it should be deployed. There are many aspects to consider including.

- Do we encourage mass vaccination across the HRA and Edge Area or target it only at hotspot areas or only at high-risk herds?
- How will the effect of the vaccine at individual level translate to the herd level in terms of reducing herd incidence, the number of reactor animals, and the duration of breakdowns?
- What are the trade impacts?
- How to identify and track vaccinated animals to ensure the correct test is used and vaccinated animals are traceable for trade purposes?

To address these issues work is beginning on three interlocking projects looking at.

1. the logistics of deployment,
2. modelling and evaluating deployment and.
3. considering the policy, legal and trade aspects of deployment.

All of this work will include dialogue with farmers.

### Molecularly DEFINED TUBERCULIN (MDT)

In addition to the DIVA skin test, APHA are also working on an alternative to Tuberculin using a defined combination of key antigens from *M.bovis*. The goal is to have a more standardised reagent than Tuberculin A and B. This should reduce between batch variation of test reagents and thus improve the quality and consistency of TB testing. The test in development contains the three antigens in the DST-F plus a further five antigens. This would be a different test to the DIVA and is intended for use outside of a BCG vaccination programme. Results have shown that MDT performs better than the DST-F DIVA skin test in infected animals with high specificity in MAP vaccinated and unvaccinated animals and it also functions well in interferon-gamma release assays [10].

### Targeted badger culling

Badger culling has been deployed over the majority of the HRA and a large part of the Edge Area. This has been delivered and largely paid for by industry. The current culling policy will be phased out with badger vaccination becoming the preferred approach. There may be a role for smaller scale targeted culls in areas where badgers are a particular problem. This may follow aspects of the Low-Risk area culling approach.

The LRA culling policy was developed in response to a TB hotspot in Cumbria. In 2015–16 there was a cluster of unusual breakdowns in a small area (~ 200km^2^) in Cumbria in the LRA not linked to cattle movements and with a unique *M.bovis* genotype, 17z, in all infected herds [11]. This genotype appears to have originated in Northern Ireland but was suspected to be circulating in badgers in this part of Cumbria. The specific cattle movement that introduced 17z to Cumbria has not been identified. Extra cattle measures were applied [12] and a survey of found dead badgers and deer was initiated in 2016. In 2017, 3 of 55 found dead badgers were *M.bovis* positive, all with 17z and all in the central part of the cluster of cattle cases. In 2018 a new policy was adopted of industry led culling in the LRA with close support from government to try and remove infection from the area. A local ownership group was formed with farmers to discuss, fund and improve biosecurity.

In the first cull in 2018 18% of badgers in the central “Minimum Infected Area” were infected, all with 17z whereas only 3% of badgers in the outer “buffer” area (Table 1) were infected. In subsequent culls [13] the prevalence of disease dropped in both areas and the buffer area switched to vaccination in 2020. Cattle incidence in the area has reduced and the 17z genotype appears to have been eliminated. The additional cattle measures are now slowly being relaxed as the area is transitioning to 12 monthly testing. Another cull area in the LRA in Lincolnshire started in 2020 again TB prevalence in badgers has declined between the first and second cull (Table 2).

**Table 1:** Prevalence of Tuberculosis in culled badgers in cull area 32-Cumbria

	Minimum Infected Area	Buffer area
2018	21%	2%
2019	14%	0%
2020	0%	No culling-badger vaccination
2021	0%	No culling- badger vaccination

**Table 2:** Prevalence of Tuberculosis in culled badgers in cull area 54-Lincolnshire

	Minimum infected area	Buffer area
2020	30%	3%
2021	18%	2%

### Challenges for targeted culling

- How much of the LRA approach in Cumbria will be applicable to HRA/Edge areas which have culled for four or more years but where culling will have ceased, and some areas will have been vaccinating?
- How to identify emerging problem areas linked to badgers in areas of higher background incidence in cattle?
- What combination of cattle epidemiology, WGS from cattle and badgers, and badger surveillance should be required?
- How to carry out disease surveillance in badgers cost effectively?

Our stakeholder group, the TB partnership, has been considering the high-level principles around this issue. We also will be trialling new approaches to badger testing this year.

## Conclusion

How could the innovations discussed today improve the TB control system?

**Fig. 5:**
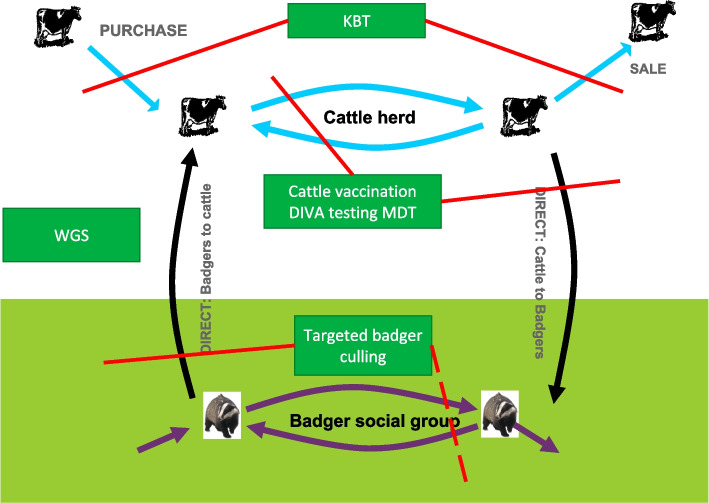
How innovations can disrupt (red lines) the spread of *M.bovis*

Returning to the concept of the *M.bovis* system all of the innovations discussed here have the potential to reduce transmission by disrupting the *M.bovis* system (Fig. 5). KBT should reduce infection moving between herds. While cattle vaccination should prevent spread within herds and protect cattle from infection from badgers. MDT should improve testing in unvaccinated herds. Targeted badger culling could provide an alternative method to controlling the disease in badgers in addition to badger vaccination. Finally, WGS should increase our understanding of all aspects of the epidemic.

## Data Availability

Data sharing is not applicable to this article as no datasets were generated or analysed for this article.

## Abbreviations

APHA: Animal and Plant Health Agency
BCG: Bacillus Calmette-Guerin
Defra: Department for Environment Food and Rural Affairs
DIVA: Detect Infected animals among Vaccinated Animals
DST-F: DIVA Skin Test Fusion
GB: Great Britain
GIS: Geographic Information System
HRA: High Risk Area
KBT: Knowledge Based Trading
LRA: Low Risk Area
MAP: Mycobacterium avium paratuberculosis
MDT: Molecularly Defined Tuberculin
OTF: Officially TB Free
OTFw: Officially TB Free status Withdrawn
TB: Tuberculosis
VMD: Veterinary Medicines Directorate
VNTR: Variable Number Tandem Repeat
WGS: Whole Genome Sequencing
WOAH: World Organisation for Animal Health
